# 

**Published:** 1892-09

**Authors:** 


### Society Meetings.

THE American Association of Obstetricians and Gynecologists will hold its fifth annual meeting at the Lindell Hotel, St. Louis, Tuesday, Wednesday, and Thursday, September 20, 21, and 22, 1892. The President, Dr. A. Vander Veer, of Albany, N. Y., wishes it understood that all members of the medical profession interested in the subjects discussed, or who are friends of the association, even though not especially interested in its branch of work, are most cordially invited to attend the several sessions.

The Lindell Hotel will be the headquarters of the Association during the meeting, and has a convention hall which will provide ample accommodations for its sessions.

By order of the Executive Council.

---

THE second International Dermatological Congress will be held in Vienna from the 5th to the 10th of September, 1892. It is expected that this will be one of the most notable dermatological meetings ever held. The congress will be presided over by Professor Kapesi, and many members of the American Dermatological Association and of the New York Dermatological Society are arranging to attend. Dr. Prince A. Morrow, of New York, is Secretary for North America.

---

THE American Orthopedic Association will hold its sixth annual meeting at the New York Academy of Medicine, under the presidency of Dr. Benjamin Lee, of Philadelphia, September 20, 21, and 22, 1892. An excellent programme has been issued, and the meeting cannot fail to prove useful and entertaining.

---

THE American Dermatological Association announces its sixteenth annual meeting, to be held at the Pequoit House, New London, Conn., September 13, 14, and 15, 1892, under the presidency of Dr. E. B. Bronson, of New York. The excellent classified programme already issued indicates an unusually interesting meeting.

---

THE American Gynecological Society will hold its regular annual meeting, in Brooklyn, September 20, 21, and 22, 1892, under the presidency of Dr. John Byrne.

### BOOKS AND PAMPHLETS RECEIVED.

Hydrotherapy of Saratoga. A Treatise on Natural Mineral Waters. By J. A. Irwin, M. A., Cambridge, Eng.; M. A., M. D., Dublin University; L. M., College of Physicians, Ire.; Member of Royal College of Surgeons, Eng.; Member of the British Medical Association; Fellow of the London Obstetrical Society, and the New York Academy of Medicine, etc.; Formerly House Surgeon Royal Free Hospital, London; Medical Officer Shropeshire and Montgomeryshire Counties Lunatic Asylum, and Physician to the Manchester Southern Hospital for Women and Children. Cassell Publishing Co., New York.

Essentials of Medical Diagnosis, arranged in the form of questions and answers, prepared especially for students of medicine, by Solomon Solis-Cohen, M. D., Professor of Clinical Medicine and Applied Therapeutics in the Philadelphia Polyclinic; one of the Physicians to the Philadelphia Hospital, etc.; and Augustus A. Eshner, M. D., Instructor in Clinical Medicine in Jefferson Medical College and in the Philadelphia Polyclinic; Registrar in the Neurological Department of the Philadelphia Hospital, etc., with fifty-five illustrations, some of which are colored, and a frontispiece. Philadelphia: W. B. Saunders, 913 Walnut street. 1892.

A New Pronouncing Dictionary of Medicine. Being a voluminous and exhaustive hand-book of Medical and Scientific Terminology, with phonetic pronunciation, accentuation, etymology, etc. By John M. Keating, M. D., LL.D., Fellow of the College of Physicians of Philadelphia; Vice-President of the American Pediatric Society; Ex-President of the Association of Life Insurance Medical Directors; formerly Visiting Obstetrician to the Philadelphia Hospital (Blockley), and Lecturer on the Diseases of Women and Children; Consulting Physician for the Diseases of Women, St. Agnes' Hospital, Philadelphia; Gynecologist to St. Joseph's Hospital, Philadelphia; Editor Cyclopedia of the Diseases of Children, etc.; and Henry Hamilton, author of a New Translation of Virgil's Aeneid into English Rhyme; co-author of Saunders' Medical Lexicon, etc., with the collaboration of J. Chalmers DaCosta, M. D., and Frederick A. Packard, M. D. With an appendix containing important tables of bacilli, micrococci, leucomaines, ptomaines, drugs, and materials used in antiseptic surgery; poisons and their antidotes; weights and measures; thermometric scales; new officinal and unofficinal drugs, etc., etc. Philadelphia: W. B. Saunders, 913 Walnut street. Price, $5 net, cloth; $6 net, sheep.

Transactions of the American Dermatological Association at its fifteenth annual meeting, held at the Shoreham Hotel, Washington, D. C., on the 22d, 23d, 24th, and 25th of September, 1891, in connection with the Congress of American Physicians and Surgeons. Official report of the proceedings. By George Thomas Jackson, M. D., Secretary. New York, 1891.

U. S. Department of Agriculture. Division of Chemistry. Bulletin No. 13. Foods and Food Adulterants. Investigations made under the direction of H. W. Wiley, Chief Chemist. Part Seventh. Tea, Coffee, and Cocoa Preparations. By Guilford L. Spencer, Assistant Chemist, with the collaboration of Mr. Ervin E. Ewell. Published by authority of the Secretary of Agriculture. Washington: Government Printing Office. 1892.

U. S. Department of Agriculture, Division of Chemistry, Bulletin No. 33. Experiments with Sugar Beets, in 1891, by Harvey W. Wiley, Chief Chemist of the U. S. Department of Agriculture, and Director of the Department Sugar Experiment Stations at Schuyler, Nebraska, etc., with the collaboration of Dr. Walter Maxwell, Prof. W. A. Henry, and others. Published by authority of the Secretary of Agriculture. Washington: Government Printing Office. 1892.

U. S. Department of Agriculture, Division of Chemistry, Bulletin No. 34. Record of Experiments with Sorghum, in 1891, by Harvey W. Wiley, Chemist of the U. S. Department of Agriculture, and Director of the Department of Sugar Experiment, etc., with the collaboration of Dr. G. L. Spencer, Mr. A. A. Denton, and Mr. Wibray J. Thomson. Published by authority of the Secretary of Agriculture. Washington: Government Printing Office. 1892.

What to Do in Case of Accident. By B. Merrill Ricketts, Ph. B., M. D., Cincinnati. Printed by James Barclay, 124 Canal street. 1892.

Diseases of Women: a Manual of Non-Surgical Gynecology. Designed especially for the use of students and general practitioners. By F. H. Davenport, A. B., M. D., Instructor in Gynecology, Harvard Medical School; Assistant Surgeon to the Free Hospital for Women; Physician to the Department of Gynecology, Boston Dispensary. Second edition, revised and enlarged, with numerous illustrations. Philadelphia: Lea Brothers & Co. 1892.

Cardiac Outlines for Clinical Clerks and Practitioners, and First Principles in the Physical Examination of the Heart, for the Beginner. By William Ewart, M. D., Cantab., F. R. C. P. Physician to St. George's Hospital; Clinical Lecturer and Teacher of Practical Medicine in the Medical School; Physician to the Belgrave Hospital for Children; Additional Examiner, in 1891, for the third M. B. of the University of Cambridge; late Assistant Physician and Pathologist to the Brompton Hospital for Consumption and Diseases of the Chest. With sixty-two illustrations. G. P. Putnam's Sons, New York, 27 West Twenty-third street. London, 24 Bedford street, Strand. The Knickerbocker Press. 1892.

A Treatise on Diseases of the Nose and Throat, in two volumes. By Francke Huntington Bosworth, A. M., M. D., Professor of Diseases of the Throat in the Bellevue Hospital Medical College, New York; Consulting Laryngologist to the Presbyterian Hospital; Consulting Physician to the O. D. P. of the Bellevue Hospital; Fellow of the American Laryngological Association, of the American Climatological Association, of the New York Academy of Medicine; Member of the New York Laryngological Society, of the Medical Society of the County of New York, etc., etc. Volume II. Diseases of the Throat. With three colored plates and 125 wood-cuts. New York: William Wood & Co. 1892.

Bureau of Education. Circular of Information No. 6, 1891. Contributions to American Educational History, edited by Herbert B. Adams. No. 15, History of Higher Education in Massachusetts, by George Gary Bush, Ph. D. Washington: Government Printing Office. 1891.

Proceedings of the Florida Medical Association. Session of 1892. Jacksonville, Fla.: Dacosta Printing and Publishing House. 1892.

Transactions of the thirteenth annual meeting of the American Laryngological Association, held in the city of Washington, D. C., September 22, 23, and 24, 1891. New York: D. Appleton & Company. 1892.

Transactions of the Medical Society of the State of New York for the year 1892. Edited by Frederlc C. Curtis, M. D., Secretary. Octavo, pp. viii.—540. Published by the Society. William J. Dornan, Printer.

Book on the Physician Himself, and Things that Concern His Reputation and Success. By D. W. Cathell, M. D. New tenth edition (author's last revision). Thoroughly revised, enlarged, and rewritten. In one handsome royal octavo volume. 348 pages. Bound in extra cloth. Price, post-paid, $2.00 net. Philadelphia: The F. A Davis Co., Publishers, 1231 Filbert street.

Bureau of Education. Circular of Information No. 1, 1892. Southern Women in the Recent Educational Movement in the South. By Rev. A. D. Mayo, M. A. Washington: Government Printing Office. 1892.

Transactions of the eighth annual meeting of the American Climatological Association, held at Washington, D. C., September 22, 23, 24, and 25, 1892. 8vo, pp. xvi.—276. Philadelphia: W. B. Saunders. 1892.

Diseases of the Stomach. By Dr. C. A. Ewald, Extraordinary Professor of Medicine at the University of Berlin, Director of the Augusta Hospital, etc. Authorized Translation from the Second German Edition, with Special Additions by the Author. By Morris Manges, A. M., M. D., Attending Physician to Out-door Department, Mount Sinai Hospital, New York, etc. With 30 illustrations, 8vo, pp. xiv.—497.

Naphey's Modern Therapeutics. Medical and Surgical, including the Diseases of Women and Children. A Compendium of Recent Formula and Therapeutical Directions from the Practice of Eminent Contempory Physicians, American and Foreign. Ninth edition, revised and enlarged. Vol. I. General Medicine and Diseases of Children, by Allen J. Smith, M. D., Assistant Demonstrator of Morbid Anatomy and Pathological Histology, Lecturer on Urinology, etc., University of Pennsylvania, and J. Aubrey Davis, Assistant Demonstrator of Obstetrics, etc., University of Pennsylvania, Octavo, pp. xx.—1,034. Philadelphia: P. Blakiston, Son & Company. Buffalo: Peter Paul & Brother.

Notice to Contributors.—We are glad to receive contributions from every one who knows anything of interest to the profession. Articles designed for publication in the Journal should be handed in before the first day of the month. The Editors are not responsible for the views or opinions of contributors. All communications should be addressed to the Managing Editor, 284 Franklin St., Buffalo, N. Y.

